# Machine learning algorithms for predicting undernutrition among under-five children in Ethiopia

**DOI:** 10.1017/S1368980021004262

**Published:** 2022-02

**Authors:** Fikrewold H Bitew, Corey S Sparks, Samuel H Nyarko

**Affiliations:** Department of Demography, College for Health, Community and Policy, The University of Texas at San Antonio, 9947 Bricewood Hill, San Antonio, TX 78254, USA

**Keywords:** Predictive algorithms, Determinants, Child undernutrition, Spatial variations, Machine learning, Ethiopia

## Abstract

**Objective:**

Child undernutrition is a global public health problem with serious implications. In this study, we estimate predictive algorithms for the determinants of childhood stunting by using various machine learning (ML) algorithms.

**Design:**

This study draws on data from the Ethiopian Demographic and Health Survey of 2016. Five ML algorithms including eXtreme gradient boosting, k-nearest neighbours (k-NN), random forest, neural network and the generalised linear models were considered to predict the socio-demographic risk factors for undernutrition in Ethiopia.

**Setting:**

Households in Ethiopia.

**Participants:**

A total of 9471 children below 5 years of age participated in this study.

**Results:**

The descriptive results show substantial regional variations in child stunting, wasting and underweight in Ethiopia. Also, among the five ML algorithms, xgbTree algorithm shows a better prediction ability than the generalised linear mixed algorithm. The best predicting algorithm (xgbTree) shows diverse important predictors of undernutrition across the three outcomes which include time to water source, anaemia history, child age greater than 30 months, small birth size and maternal underweight, among others.

**Conclusions:**

The xgbTree algorithm was a reasonably superior ML algorithm for predicting childhood undernutrition in Ethiopia compared to other ML algorithms considered in this study. The findings support improvement in access to water supply, food security and fertility regulation, among others, in the quest to considerably improve childhood nutrition in Ethiopia.

Undernutrition is a serious global public health problem, which results in high mortality and overall disease burden^(1)^ and is common among under-five children, particularly in low and middle-income countries^(1,2)^. Even though global rates have declined, undernutrition rates remain high among children in sub-Saharan Africa^(3,4)^, with Eastern Africa having one of the highest stunting rates (exceeding 30 %)^(5)^, including Ethiopia^(6)^. In Ethiopia, undernutrition in the form of under-five stunting (low height for age) decreased from 58 % in 2000 to 38 % in 2016, a reduction of about one-third. Besides, under-five underweight (low weight-for-age) declined from 41 % to 24 % during the same period^(6–9)^. Despite these achievements which followed an improvement in food security due to several government policy interventions^(10)^, undernutrition among children remains very high making it difficult to achieve Ethiopia’s commitment to the Seqota Declaration of ending child undernutrition by 2030^(11)^. This may be caused by a myriad of factors including population pressure, drought, disease outbreak, chronic poverty, pre- and post-harvest crop losses^(12)^ as well as increasing food prices^(13)^ which constrain food security and nutritional status in the country.

Meanwhile, several studies have examined the spatial variations and determinants of undernutrition among under-five children in Ethiopia based on the traditional analytical approach^(9,14–16)^. Most of these studies focussed only on specific parts of the country such as rural parts of Tigray and Somali regions, or are limited to specific localities^(17)^ which are not nationally representative. The few studies^(18,19)^ that show evidence on the spatial variations in undernutrition among children in Ethiopia mainly focussed on stunting and overlooked other indicators of child undernutrition, such as wasting and underweight.

Furthermore, machine learning (ML) is a powerful approach that intersects artificial intelligence and statistical learning in the process of discovering unknown relationships or patterns^(20)^. Modern ML algorithms have shown superior predictive ability in addressing classification problems when compared with classical statistical models. Various ML algorithms have been applied in medical research^(21–24)^. For instance, ML algorithms such as random forest (RF), support vector machine and artificial neural networks have been used to predict the status of diseases such as acute appendicitis and diabetes^(21,22)^. A related study in Bangladesh has shown that the RF algorithm was superior to other ML algorithms such as linear discriminant analysis, k-nearest neighbours (k-NN), support vector machines and logistic regression^(25)^. Moreover, a study in Nigeria used Bayesian Additive Regression Trees to show that maternal education decreases severe child undernutrition when mothers acquire 10 years of education or higher^(26)^. Nevertheless, a scoping review conducted by Kino *et al.* ^(27)^ has shown that among the huge volumes of social determinants of health studies published annually, only a few used ML techniques, which creates the opportunity to conduct this research further. As well, most of these ML studies used United States data and, therefore, provides a direction to explore public health concerns from other parts of the world^(27)^. As a corollary, in this study, we used various ML algorithms that were not extensively used in previous studies to predict child undernutrition determinants in Ethiopia.

Ultimately, a comparison of five ML algorithms was illustrated for three indicators of child undernutrition (stunting, wasting and underweight). The study initially presented a spatial map for under-five nutritional status in Ethiopia to provide an overview of child undernutrition disparities across the regions of the country. The main goal of this study is to provide evidence on the best predictive algorithm for child undernutrition risk factors in Ethiopia. This study will provide much understanding of how the various indicators of child undernutrition vary with space and the risk factors that underlie these variations, which would be necessary for targeting programs and interventions given the limitation of resources in the country.

## Methods

### Data source

This study uses data from the 2016 Ethiopian Demographic and Health Survey. The 2016 Ethiopian Demographic and Health Survey is currently the latest and part of the world demographic and health survey series that is conducted every 5 years. It is a nationally representative household survey that collects data on a broad range of population and health issues to enhance maternal and child health in Ethiopia^(6)^. The Ethiopian Demographic and Health Survey survey used a multi-stage stratified sampling procedure to select respondents from households in a total of 624 clusters^(6)^. The study sample is limited to 9471 children below age five. This was based on retrospective information obtained from mothers about the BMI of their children within the 5 years preceding the survey (2011–2016).

### Study variables and measurements

The outcomes of interest in this study are under-five stunting, wasting and underweight status. *Z*-scores of anthropometric measurements – height-for-age (stunting), weight-for-age (underweight) and weight-for-height (wasting) – were used to evaluate the nutritional status. According to WHO, undernutrition indicators are determined by the following standard measures: stunting: height-for-age < –2 sd; wasting: weight-for-height < –2 sd and underweight: weight-for-age < –2 sd of the WHO Child Growth Standards median^(28,29)^. Severe stunting, wasting and underweight were those children whose height-for-age, weight-for-height and weight-for-age *Z*-score below minus 3 (−3) sd. This study, thus, considered all three undernutrition indicators to predict childhood undernutrition determinants. In this regard, the outcomes were binary coded as 1 for stunted, wasted and underweight if the standard was met else 0 for not stunted, not wasted and not underweight. A set of covariates were considered as the possible risk factors for childhood undernutrition in Ethiopia (See Appendix). In the ML algorithms, we incorporated as many variables as possible from the DHS which have less percentage of missing data. Essentially, the only variables excluded from the study were those that have more than 50 % missing data due to their impact on the performance of the algorithms.

### Analytic strategy

The R programming language (version 3.6.0)^(30)^ and the R packages caret^(31)^ and caretEnsemble^(32)^ were used to perform the data processing and analysis. Five ML algorithms (xgbTree, generalised linear model (GLM), NNet, RF, k-NN) were applied to determine the predictive power of ML algorithms and to identify the top-20 most important determinants for each of childhood undernutrition indicators (stunting, wasting and underweight).

### Logistic regression

The binomial GLM is typically used to analyse binary data and is commonly used as an inferential tool in population health research, but it also can be used as a binary classification algorithm. No tuning is needed for GLM because the algorithm has no hyperparameters and assumes a logit relationship between response and predictors.

### Random forest

RF is a supervised ensemble learning method that acts based on decision trees^(33)^. RF algorithm repeatedly samples the variables in the training data set many times, each time using a random set of predictor variables to produce a regression classification tree. After many of these trees are formed, the predictive performance of each variable is measured, and the best set of variables is obtained. It is very flexible and fast that can be used for both classification and regression.

### Extreme gradient boosting

xgbTree is a scalable ensemble technique that has been demonstrated to be a reliable and efficient ML challenge solver^(34)^. The xgbTree is chosen because it uses an efficient and scalable implementation of the gradient boosting framework and supports various objective functions, including regression, classification and ranking^(35)^. It has better control against overfitting by using more regularised algorithm formalisation, in comparison to prior algorithms. It has a high rate of success in Kaggle competitions, particularly for structured features^(36)^.

### Neural networks

Neural networks represent a method of statistical learning based on the model of neurons in the brain. In some sense, they can be thought of as nonlinear regression based on how the observed data can affect the outcome. Visually, however, they can be seen as layers of inputs and outputs. Weighted combinations of the inputs are created and put through a function (e.g. the sigmoid function) to produce the next layer of inputs^(37)^. The next layer goes through the same process to produce either another layer or to predict the output, which is the final layer. All the layers between the input and output are usually referred to as ‘hidden’ layers. Some of the strengths include having good prediction generally, incorporating the predictive power of different combinations of inputs and having tolerance for correlated inputs^(37)^.

### k-nearest neighbours

k-NN is a robust and adaptive classification algorithm that is part of the supervised ML family. It is a non-parametric algorithm that does not rely on any strict assumptions about the underlying data. The decision boundary of the algorithm depends on a few input points and their particular positions. Thus, the classification of new cases is based on a similarity or the use of observations in the training set that are closest in metric space^(38)^.

### ML approach

Following the standard methods for ML techniques, the data were split into two sets (training and testing) to learn from the data, train the classification algorithms and identify patterns within the data. Once the algorithms were trained, they were applied to the test dataset, and algorithm accuracy was assessed. The data were trained twice – with (60 % train, 40 % test) and (70 % train and 30 % test) – but a reasonable outcome was observed in the widely used classification of 70 % train and 30 % test. Thus, the training set consisted of 70 % of the observed data while the remaining 30 % of the cases were held out as a test or validation set. Five ML algorithms (xgbTree, GLM, NNet, RF, k-NN) were applied by using a sample of 70 % of the individuals in each group (training data set, *n* 5147) and validated in the remaining 30 % (test data set, *n* 1716). Missing cases were then disposed of while running the ML algorithms. All algorithms were trained based on 10-fold cross-validation. We used 10-fold cross-validation on the training set, and the performance was estimated on the testing set.

### Combining algorithms into ensemble predictions

To increase the accuracy of the algorithms, we used ‘Stacking’, the most popular method for combining the predictions from different algorithms. Using ‘Stacking’, multiple algorithms (typically of differing types) can be built and a supervisor algorithm that learns how to best combine the predictions of the primary algorithms be generated^(38)^. Thus, in this study, the predictions of the selected caret algorithms (xgbTree, GLM, NNet, RF, k-NN) were combined using stacking.

### Algorithm evaluation

To verify the algorithm’s performance in terms of classifications, a confusion matrix (also known as an error matrix) is used. A confusion matrix of a binary classification is a two-by-two table showing values of True Negatives, False Negatives, True Positives and False Positives resulting from predicted classes of data. The confusion matrix allows the measures of rates such as prediction accuracy, sensitivity and specificity^(39)^.

#### Accuracy

Accuracy is the basis of estimating the performance of any predictive algorithm. It estimates the ratio of right predictions to the total number of data points evaluated. This study was comprised of the best accuracies that were obtained by several ML algorithms after applying the feature selection as well as k-fold techniques.

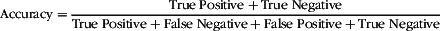




#### Sensitivity

Sensitivity is the proportion of real positive cases that got predicted as positive (or true positive). It is also termed *recall*. This implies that there will be another proportion of real positive cases, which would get predicted incorrectly as negative (termed as the *false negative*). This can also be presented in the form of a false-negative rate.

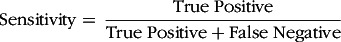




#### Specificity

Specificity is the proportion of real negative cases that got predicted as the negative (or true negative). This implies that there will be another proportion of real negative cases, which would get predicted as positive and could be termed as *false positives*. This can also be presented in the form of a false-positive rate.

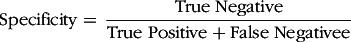




### Cohen’s *κ*

The *κ* statistic (or value) is a metric that compares an Observed Accuracy with an Expected Accuracy (random chance). The *κ* statistic is used not only to evaluate a single classifier but also several classifiers amongst themselves. The calculation of the Observed Accuracy and Expected Accuracy is important for the comprehension of the statistic which is usually illustrated using a confusion matrix. Landis and Koch^(40)^ provide the following to measure the values of this statistic: 0 indicates *no agreement*, 0–0·20 as slight, 0·21–0·40 as fair, 0·41–0·60 as moderate, 0·61–0·80 as substantial and 0·81–1 as almost perfect.

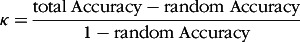




Total accuracy is simply the sum of true positive and true negatives, divided by the total number of items, that is:

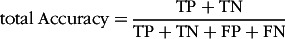




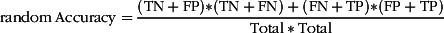




## Results

### Descriptive results

Out of the 9471 children below 5 years in the study sample, 38·4 % of them were reported to be stunted, 10 % were wasted and 23·3 % were underweight. Close to half of the children (46·6 %) experienced some form of malnutrition (were either stunted, wasted or underweight). About half of the children (50·4 %) were aged less than 30 months, and the majority (64·6 %) belonged to mothers aged less than 20. More than half of the children (51·9 %) were males. Two-third (67·2 %) of these children were born at home, with the remaining children (32·8 %) being born in health facilities. About 46 % of the children were from poor households, while 89 % resided in rural settings. The majority were at least third-order births (65·4 %) and 2–4 years interval births (55·8 %). Also, about 44 % of the children did not have access to an improved water source while about 91 % of them had no access to improved toilet facilities. Further, about 45 % of them were children of mothers with two children (parity 2) while 17·4 % of them were children of mothers with three or more children (Table not shown).

### Spatial distribution of childhood undernutrition indicators

**Fig. 1:**
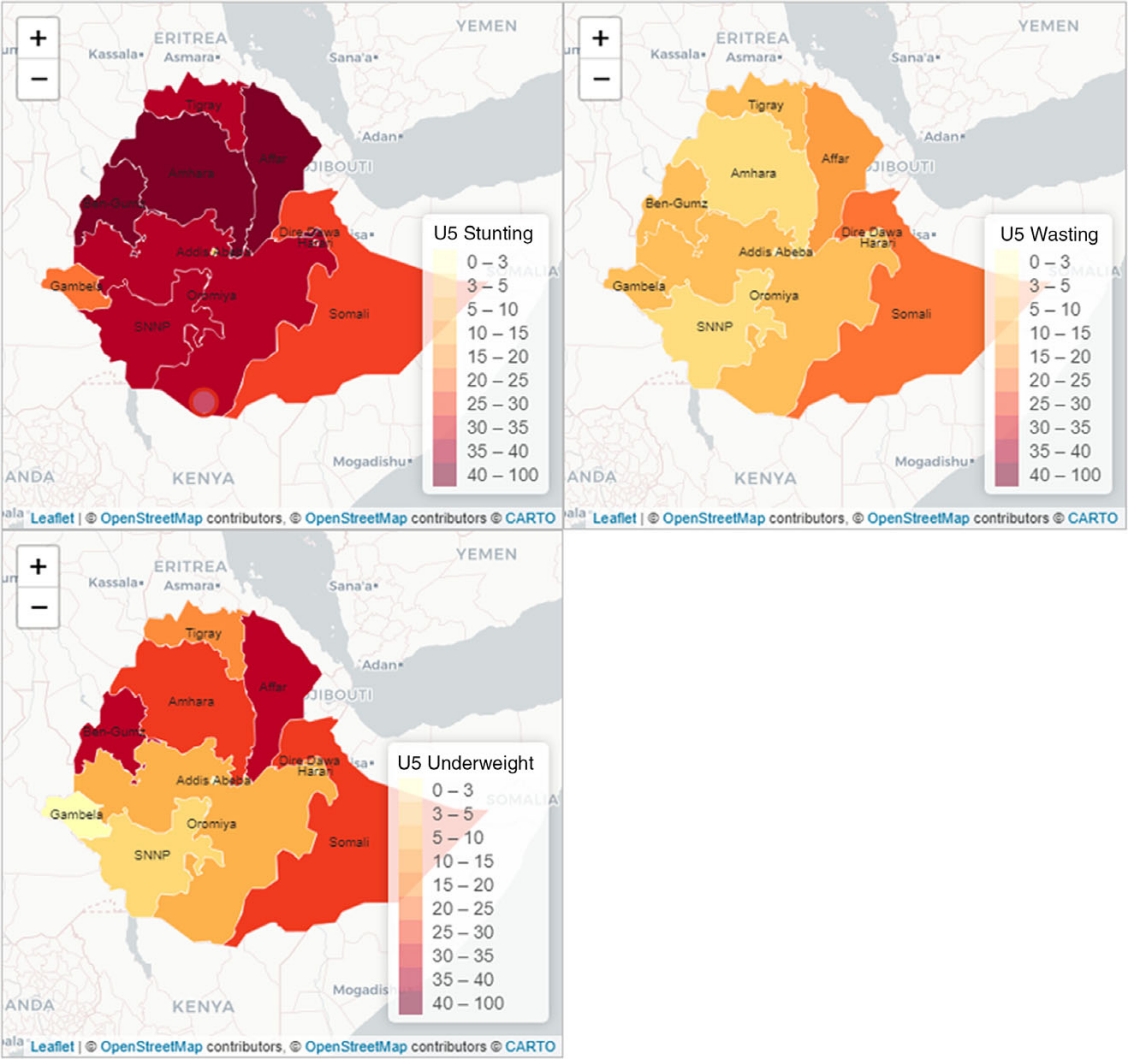
Spatial variations in undernutrition indicators by administrative regions in Ethiopia, EDHS, 2016. Source: Created by the authors based on 2016 EDHS

**Fig. 2:**
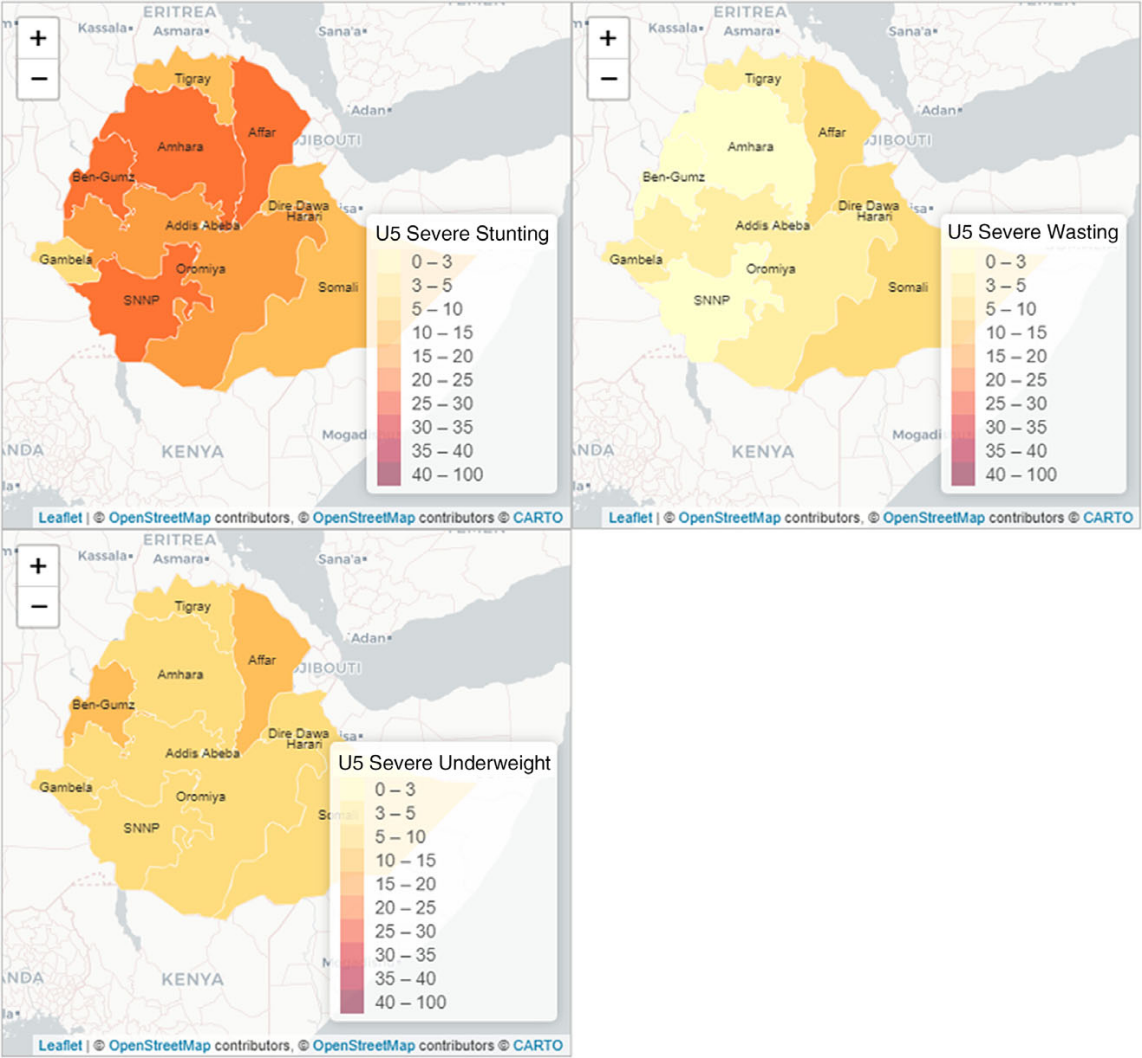
Spatial variations in severe undernutrition indicators by administrative region in Ethiopia, EDHS, 2016. Source: Created by the authors based on 2016 EDHS

Figure 1 presents a visualisation of the spatial variations of the three childhood undernutrition outcomes. The results show considerable regional variations in stunting, wasting and underweight as measures of undernutrition in the country. It is visually clear that Amhara, Benishangul-Gumuz, Affar and Dire Dawa regions were the most affected by stunting with Gambela and Somali being the least affected regions. Wasting was most prevalent in the eastern part of the country, comprising of the Somali and Affar regions and followed by Gambella and Benishangul-Gumuz, among others in the west. Amhara and Southern Nations, Nationalities and Peoples (SNNP) regions were, however, least affected by wasting. Underweight was most prevalent in the Affar region in the northeast, and the Benishangul-Gumuz region in the western part of the country. However, underweight was the least prevalent in the Gambella region. Severe stunting, wasting and underweight showed similar patterns of variations even though at comparatively lower levels (Fig. 2).

### Predictive algorithms for child undernutrition indicators

#### Stunting

**Fig. 3:**
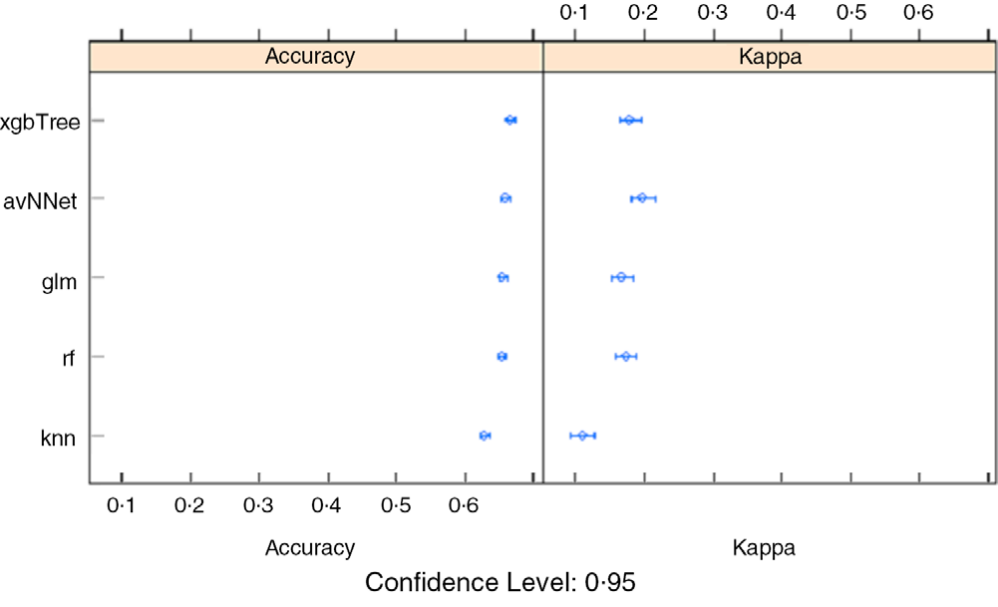
Stunting: comparison of sub-algorithms for stacking ensemble in R

The under-five stunting prediction accuracy was found to be low for all algorithms, between 62·9 and 67·7 % accuracy on the test set, although the xgbTree had the highest overall accuracy (Table 1). The xgbTree had relatively higher sensitivity, meaning that it was accurate at distinguishing the stunting cases from the non-stunted cases, but had low specificity, meaning that it was not good at discerning the non-stunting cases. More metrics show that the algorithm is relatively better at predicting both positive (stunted) and negative (no-stunted) cases. The algorithm was able to correctly identify 72 % of the stunted, which suggests that it was relatively better at predicting the stunted cases. The GLM algorithm showed slightly lower accuracy (65·5 %), compared to xbgTree but higher than other ML algorithms (Table 1, Fig. 3).

**Table 1:** Performance indicators of all the five machine learning algorithms

Stunting	k-Nearest neighbours (k-NN)		Neural network (NNet)		Logistic regression algorithm (GLM)		Random forest algorithm (RF)		eXtreme gradient boosting (xgbTree)	
Accuracy	63·70 %	61·5 %, 65·9 %	65·0 %	62·9 %, 67·2 %	65·50 %	59·3 %, 63·7 %	65·3 %	62·2 %, 69·8 %	67·70 %	65·8 %, 69·6 %
*κ*	0·11		0·20		0·17		0·17		0·18	
Sensitivity	65·2 %		65·1 %		91·70 %		69·90 %		71·60 %	
Specificity	46·4 %		63·6 %		17·50 %		53·60 %		57·30 %	
Wasting										
Accuracy	87·79 %	86·3 %, 89·2 %	86·9 %	86·8 %, 87·0 %	87·0 %	86·2 %, 87·5 %	88·1 %	86·0 %, 90·2 %	88·0 %	86·8 %, 88·0 %
*κ*	0·01		0·00		0·02		0·00		0·01	
Sensitivity	87·79 %		87·79 %		91·70 %		88·20 %		88·20 %	
Specificity	0·0 %		0·0 %		17·50 %		54·50 %		40·00 %	
Underweight										
Accuracy	73·0 %	72·6 %, 76·5 %	74·7 %	74·6 %, 75·2 %	74·20 %	71·8 %, 76·1 %	74·80 %	72·7 %, 76·7 %	75·70 %	74·0 %, 75·9 %
*κ*	0·07		0·00		0·09		0·00		0·05	
Sensitivity	74·6 %		74·5 %		84·60 %		76·90 %		77·50 %	
Specificity	57·1 %		0·0 %		36·80 %		49·30 %		55·50 %	

#### Wasting

**Fig. 4:**
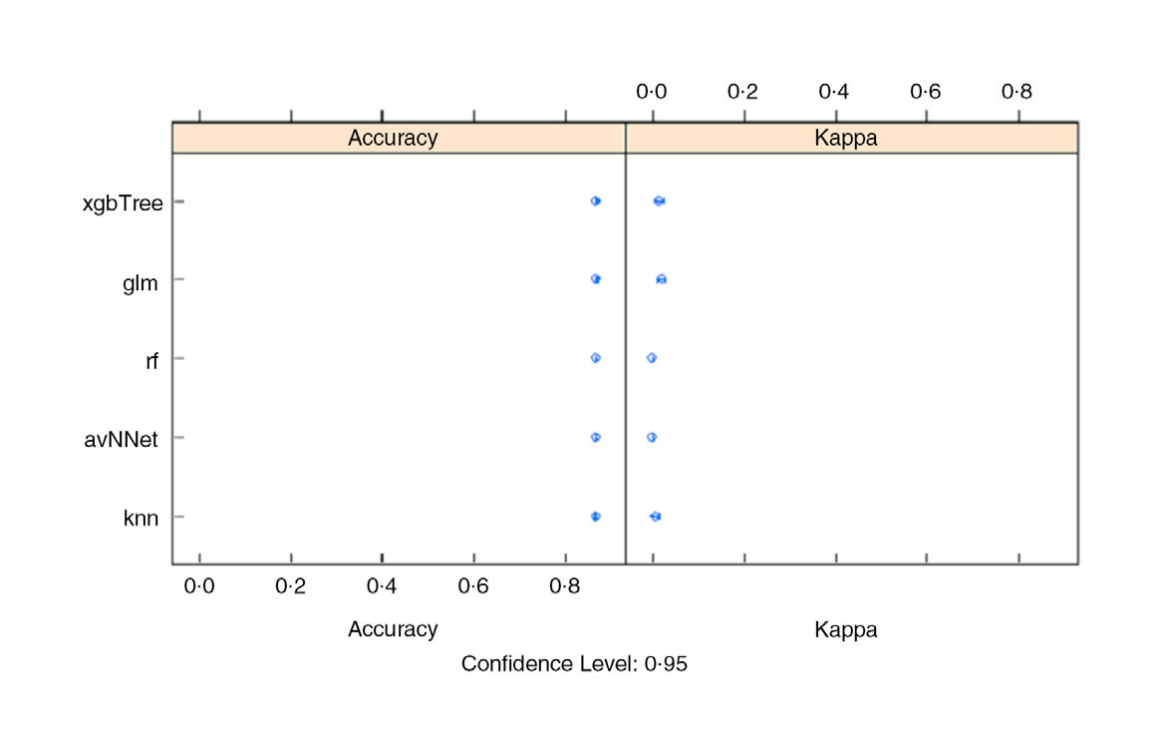
Wasting: comparison of sub-algorithms for stacking ensemble in R

The under-five wasting prediction accuracy was again found to be highest for the xgbTree with a slightly higher level of accuracy (88 %) (Table 1). Interestingly, all the selected algorithms showed more or less similar accuracy. The best predicting algorithms (xgbTree) were able to correctly identify 88·2 % of the wasted cases, which is an indication of slightly lower prediction power compared to the GLM algorithm in predicting the wasting cases. The GLM algorithm, however, showed a slightly lower overall accuracy (87·0 %) (Table 1, Fig. 4).

#### Underweight

**Fig. 5:**
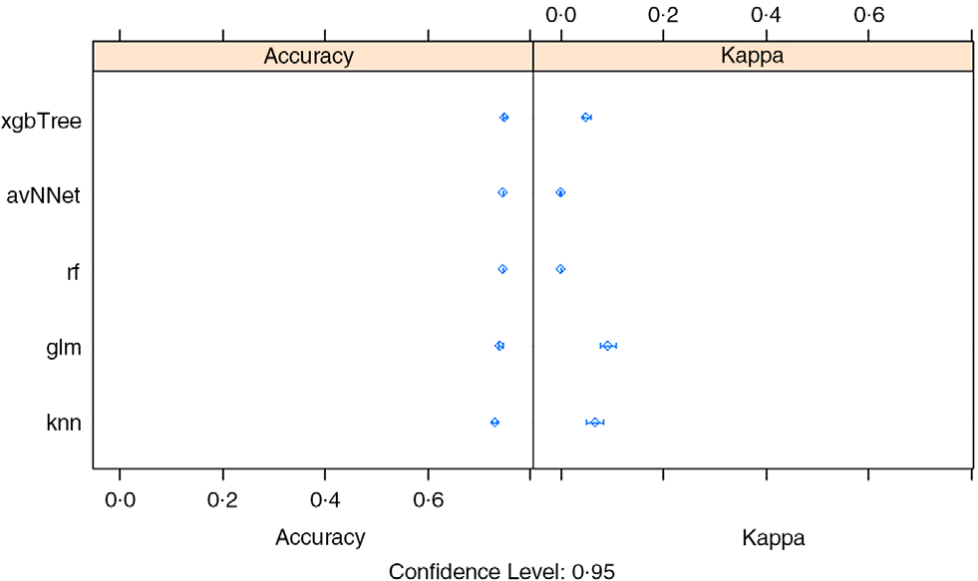
Underweight: comparison of sub-algorithms for stacking ensemble in R

As with stunting and wasting, the xgbTree algorithm was found to have the highest predictive ability (75·7 %), with a sensitivity of 77·5 % and specificity of 55·50 %. However, the k-NN algorithm indicated the lowest performance with accuracy, sensitivity and specificity of 73·0 %, 74·6 % and 57·1 %, respectively (Table 1, Fig. 5).

### The important determinants of childhood undernutrition indicators

**Fig. 6:**
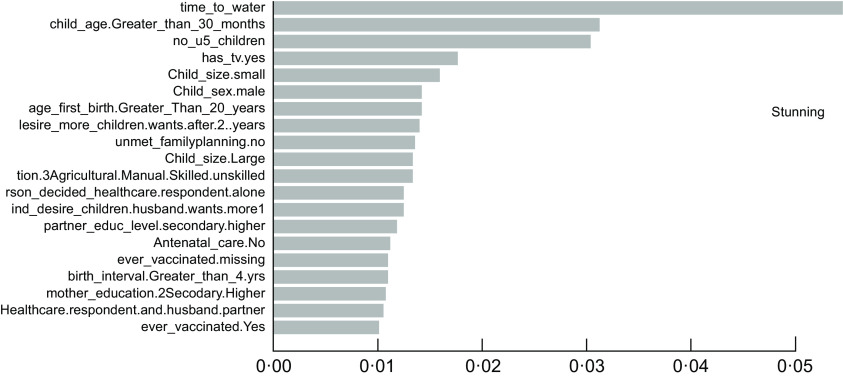
Top 20 most important variables from the xgbTree algorithm based on mean decrease Gini for stunting

**Fig. 7:**
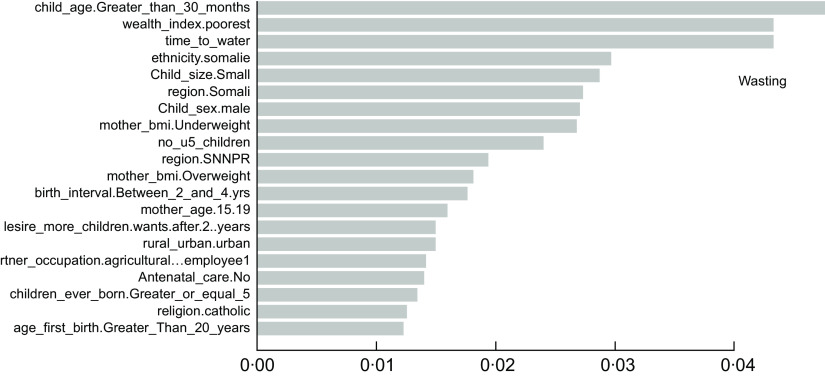
Top 20 most important variables from the xgbTree algorithm based on mean decrease Gini for wasting

**Fig. 8:**
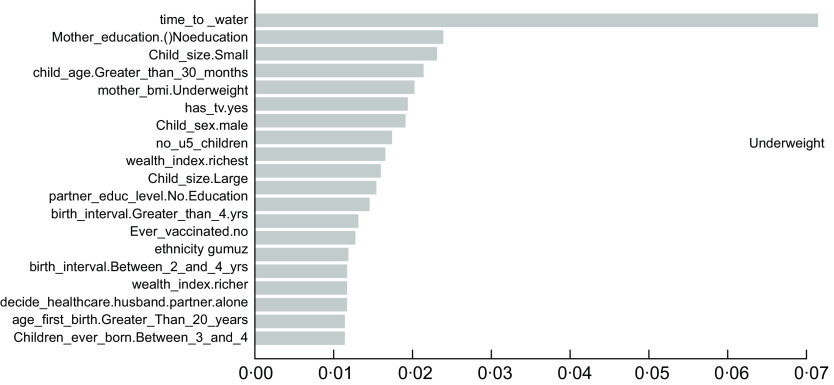
Top 20 most important variables from the xgbTree algorithm based on mean decrease Gini for underweight

As described in the above section, the accuracy results indicated that the XgbTree algorithm was the best for all the three predicting factors (stunting, wasting, and underweight), in terms of their accuracy, area under the curve – receiver operating characteristics (AUC-ROC) curve. Based on the most accurate algorithm (xgbTree), the top-20 important variables are presented out of a total number of thirty-seven variables used according to their mean decreasing Gini (Figs 6–8).

Interestingly, the top five most important among these variables were varied across all the three indicators of undernutrition. For stunting, time to water source (time_to_water), child age 30+ months (child_age_Greater_than_30_months), number of under-five children (no_u5_children), television ownership (has_tv.yes) and small birth size (child_size. Small) were the top-five important variables. For wasting, child age 30+ months (child_age_Greater_than_30_months), poorest wealth status (wealth_index.poorest), time to the water source (time_to_water), Somali ethnicity (ethnicity. Somali) and small birth size (child_size. Small) were found to be the top-five important variables. Likewise, time to the water source (time_to_water), no maternal education (mother education0.Noeducation), small birth size (child_size. Small), months (child_age_Greater_than_30_months) and maternal underweight status (mother_bmi. Underweight) were shown to be the top-five important variables predicting childhood underweight status. Time to the water source, child age 30+ months and small birth size appeared to be the common top-five important variables across the three outcomes.

## Discussion

Our descriptive findings show that there are substantial variations in all three nutritional indicators (stunting, wasting and underweight) among the regions in Ethiopia. Stunting is most prevalent among the northern regions such as Affar and Amhara, and in the western region such as Benishangul-Gumuz but least prevalent in Gambella and Somali in the south-west and south-east regions, respectively. For wasting, the prevalence is highest in the Somali region but lowest in the Amhara region. Also, underweight is most prevalent in the Affar region but least prevalent in Gambela. Evidence of similar geographical variabilities in stunting, wasting and underweight has been shown in Ethiopia^(14)^. It has been shown that food diversity and the number of meals that children eat per day play a significant role in stunting and underweight while food insecurity also has an important role to play in wasting^(41)^. Regions such as Amhara, Affar and Tigray are prevalent in food insecurity, and calorie intake per adult has been found to decrease in Beneshangul Gumuz and Amhara in recent years^(42)^. Reductions in the number of meals per day have also been shown to be common in these regions that are more frequently affected by drought and are targets of Productivity Safety Net programs^(12,43)^ despite the observed positive effects of various policy interventions on food security in some regions^(10)^. These considerable regional disparities in the nutrition indicators have profound implications for the nutritional status of under-five children in the country.

Regarding the predictive algorithms, the xgbTree algorithm appeared to have the highest predictive accuracy for all the undernutrition outcomes. It is, therefore, noteworthy that even though the traditional logistic regression algorithm (GLM) has shown the lowest predictive accuracy compared to the xgbTree and the RF, the advantage it has over the others is that its results are quite interpretable in terms of the estimated predictors in the algorithm. Similarly, a variety of ML approaches have been applied to health issues including childhood anaemia^(44)^ and nutritional status^(45)^ and have demonstrated high quality and valid predictions.

Findings from the best predicting algorithm (xgbTree) show that the key factors underlying undernutrition are diverse across the three indicators of undernutrition. Nevertheless, time to the water source, child age greater than 30 months, and small birth size appears to be the commonest important predictors across the three indicators. Water sources that can be accessed in shorter time – such as pipe-borne water – are typically located within households and usually better and safer for drinking and use. Hence, shorter or easy access to water sources has been shown to be associated with reduced risk for undernutrition particularly wasting and stunting among children^(46,47)^ while the source of drinking water is an important predictor of child nutritional status^(48)^. Furthermore, it appears that children who are 30 months old and beyond have an increased risk for all kinds of undernutrition outcomes, particularly stunting and wasting. The importance of a child’s age in predicting the undernutrition status of children is adequately documented in the literature^(47–50)^ and provides support for the findings of this study. The child size at birth also appears to play an important role in determining childhood nutritional status, with children of a small birth size being greatly disadvantaged in undernutrition risks. Similar evidence of this effect has been adequately shown in the literature^(49,51,52)^ and directly supports the findings of this study.

Furthermore, the number of under-five children in the household and television ownership has shown top-five importance for stunting alone but have been rarely documented by previous studies. Also, we find evidence of considerable disadvantage in wasting risks among children from poor households in Ethiopia. Much research in sub-Saharan Africa has shown that poor household wealth is significantly associated with child undernutrition^(49,50,53)^. Quite expectedly, poorer households may have difficulty providing sufficient nutritious food for their under-five children, which may be necessary for child growth and development. In this study, ethnic minorities such as the Somalis also emerge as one of the top five important factors for wasting risks alone even though this has seldomly been shown in the literature.

As well, the findings show that lack of maternal educational attainment proffers increased risks of childhood underweight. As such, children of educated women have considerably reduced underweight risks^(54)^, possibly because highly educated women may likely have higher access to better employment opportunities with better salaries and benefits that may help to afford good nutrition for their children. This has crucial implications for child undernutrition and further underscores the need to increase women’s education to enhance child health outcomes in developing countries^(55)^. Further, we find that children of underweight mothers have a considerable disadvantage in underweight risks. This supports the findings of myriads of studies particularly in sub-Saharan Africa^(49,54)^. This may appear unsurprising, as under-five children may likely be exposed to the same risk factors faced by their underweight mothers. The importance of the sex of children has also emerged in this study, with male children appearing to be disadvantaged in undernutrition risks than females, which directly supports the extant literature in sub-Saharan Africa^(56,57)^. However, this may seem to reflect cultural-based preferential treatments between both sexes.

The findings of this study have implications for the relevance of ML algorithms in population health research. Similarly, several studies have confirmed the usefulness of ML for population health research and policy decision making in various areas including child undernutrition^(26)^, women’s height^(58)^, CVD risks^(59)^ and mortality^(60)^ as well as defining treatment effects in epidemiological studies^(61)^ which highlights how ML is increasingly being applied to predict population health outcomes^(62)^. These findings may also be useful in bias reduction^(60)^ as ML methods can accurately quantify uncertainty when data are scarce, as can be found in sub-Saharan Africa.

It is noteworthy that this study is not without a few potential limitations. While algorithms with high representation power may have the risk of overfitting the noisy training data, algorithms with lower power may suffer from underfitting and, thus, risking failing to capture the regularity in the training data set. The underfitting problem may be usually caused by insufficient data or a high-bias algorithm (i.e. the algorithm being too simple to capture a complicated hypothesis function)^(63)^. In this study, the overall lower predictive ability observed especially in the case of stunting may reflect underfitting related to a lower study sample size. In this situation, little can be done to improve predictive power, except to gather more data (more records, more features) and/or switch algorithms by considering the previous survey years’ data (Ethiopian Demographic and Health Survey 2000–2016). As well, there is a limitation of results interpretability. Unlike the traditional logistic regression algorithm (GLM) where the population parameters generated are interpretable in terms of odds ratios and the other parameters, results from ML algorithms are mainly less interpretable as they have no parameters. Notwithstanding, the ML algorithms have been widely touted for their prediction power, and this study provides an invaluable contribution to the undernutrition literature in the context of ML.

## Conclusions

This study shows considerable regional variations in childhood undernutrition and how commonly used ML algorithms could be applied to predicting child stunting, wasting and underweight determinants in Ethiopia. The findings show that the xgbTree algorithm offers better predictive accuracy than the traditional algorithm GLM. Furthermore, the best-predicting ML algorithm has shown diverse combinations of important predictors for stunting, wasting and underweight, even though there are a few common top-five predictors among them. The algorithms may, therefore, be useful to child nutrition and other population health researchers, and aid workers among other stakeholders, particularly where large data are available. The study, thus, provides evidence on how the ML approach can be leveraged to better predict the underlying risk factors of childhood undernutrition among other population health outcomes. This may create a better understanding of a child’s nutritional status and help to develop more effective policies to advance childhood nutritional status in the country. The findings reinforce the need for committed efforts to improve upon access to potable water supply and food security, as well as the socio-economic wellbeing of women in Ethiopia. There is also the need for policies and interventions to put special focus on children of small birth size, children who are over 30 months old and children of underweight mothers.

## Supplementary material

For supplementary material accompanying this paper visit https://doi.org/10.1017/S1368980021004262. click here to view supplementary material

## References

[1] Black RE , Victora CG , Walker SP et al. (2013) Maternal and child undernutrition and overweight in low-income and middle-income countries. Lancet 382, 427–451. 23746772 10.1016/S0140-6736(13)60937-X

[2] Black RE , Allen LH , Bhutta ZA et al. (2008) Maternal and child undernutrition: global and regional exposures and health consequences. Lancet 371, 243–260. 18207566 10.1016/S0140-6736(07)61690-0

[3] Svedberg P (2006) Declining child malnutrition: a reassessment. Int J Epidemiol 35, 1336–1346. 16926213 10.1093/ije/dyl157

[4] Tzioumis E , Kay MC , Bentley ME et al. (2016) Prevalence and trends in the childhood dual burden of malnutrition in low-and middle-income countries, 1990–2012. Public Health Nutr 19, 1375–1388. 26905921 10.1017/S1368980016000276 PMC5470367

[5] UNICEF, WHO & World Bank Group (2018) *Levels and Trends in Child Malnutrition*. Geneva: World Health Organization; available at https://www.who.int/nutgrowthdb/2018-jme-brochure.pdf (accessed March 2021).

[6] Central Statistical Agency & ICF (2016) Ethiopia Demographic and Health Survey 2016. Addis Ababa, Ethiopia; Rockville, MD: CSA & ICF.

[7] Headey D (2014) An Analysis of Trends and Determinants of Child Undernutrition in Ethiopia, 2000–2011. ESSP II Working Paper 70. Washington, DC; Addis Ababa, Ethiopia: International Food Policy Research Institute (IFPRI) & Ethiopian Development Research Institute (EDRI).

[8] Lindtjørn B & Alemu T (1997) Intra-household correlations of nutritional status in rural Ethiopia. Int J Epidemiol 26, 160–165. 9126516 10.1093/ije/26.1.160

[9] Negash C , Whiting SJ , Henry CJ et al. (2015) Association between maternal and child nutritional status in Hula, rural Southern Ethiopia: a cross sectional study. PLoS One 10, e0142301. 26588687 10.1371/journal.pone.0142301 PMC4654505

[10] Van der Veen A & Tagel G (2011) Effect of policy interventions on food security in Tigray, Northern Ethiopia. Ecol Soc 16, 18.

[11] The Federal Democratic Republic of Ethiopia (2016) Implementation Plan (2016–2030) Seqota Declaration. Addis Ababa: FDRE.

[12] Endalew B , Muche M & Tadesse S (2015) Assessment of food security situation in Ethiopia: a review. Asian J Agric Res 9, 55–68.

[13] Nandy S , Daoud A & Gordon D (2016) Examining the changing profile of undernutrition in the context of food price rises and greater inequality. Soc Sci Med 149, 153–163. 26723002 10.1016/j.socscimed.2015.11.036

[14] Alemu ZA , Ahmed AA , Yalew AW et al. (2016) Nonrandom distribution of child undernutrition in Ethiopia: spatial analysis from the 2011 Ethiopia demographic and health survey. Int J Equity Health 15, 198. 27912766 10.1186/s12939-016-0480-z PMC5135763

[15] Mulugeta A , Hagos F , Kruseman G et al. (2010) Child malnutrition in Tigray, northern Ethiopia. East Afr Med J 87, 248–254. 23057267 10.4314/eamj.v87i6.63083

[16] Umeta M , West CE , Verhoef H et al. (2003) Factors associated with stunting in infants aged 5–11 months in the Dodota-Sire district, rural Ethiopia. J Nutr 133, 1064–1069. 12672920 10.1093/jn/133.4.1064

[17] Hagos S , Hailemariam D , WoldeHanna T et al. (2017) Spatial heterogeneity and risk factors for stunting among children under age five in Ethiopia: a Bayesian geo-statistical algorithm. PLoS One 12, e0170785. 28170407 10.1371/journal.pone.0170785 PMC5295674

[18] Gurmu E & Etana D (2013) Household structure and children’s nutritional status in Ethiopia. Genus 69, 113–130.

[19] Sohnesen TP , Ambel AA , Fisker P et al. (2017) Small area estimation of child undernutrition in Ethiopian woredas. PLoS One 12, e0175445. 28410435 10.1371/journal.pone.0175445 PMC5391934

[20] Alghamdi M , Al-Mallah M , Keteyian S et al. (2017) Predicting diabetes mellitus using SMOTE and ensemble machine learning approach: the Henry ford exercise testing (FIT) project. PLoS One 12, e0179805. 28738059 10.1371/journal.pone.0179805 PMC5524285

[21] Choi SB , Kim WJ , Yoo TK et al. (2014) Screening for prediabetes using machine learning algorithms. Comput Math Method Med 2014, 618976. 10.1155/2014/618976 PMC4140121 25165484

[22] Yu W , Liu T , Valdez R et al. (2010) Application of support vector machine modeling for prediction of common diseases: the case of diabetes and pre-diabetes. BMC Med Inform Decis Mak 10, 16. 20307319 10.1186/1472-6947-10-16 PMC2850872

[23] Hsieh CH , Lu RH , Lee NH et al. (2011) Novel solutions for an old disease: diagnosis of acute appendicitis with random forest, support vector machines, and artificial neural networks. Surgery 149, 87–93. 20466403 10.1016/j.surg.2010.03.023

[24] Zhao Y , Healy BC , Rotstein D et al. (2017) Exploration of machine learning techniques in predicting multiple sclerosis disease course. PLoS One 12, e0174866. 28379999 10.1371/journal.pone.0174866 PMC5381810

[25] Talukder A & Ahammed B (2020) Machine learning algorithms for predicting malnutrition among under-five children in Bangladesh. Nutrition 78, 110861. 32592978 10.1016/j.nut.2020.110861

[26] Kraamwinkel N , Ekbrand H , Davia S et al. (2019) The influence of maternal agency on severe child undernutrition in conflict-ridden Nigeria: modeling heterogeneous treatment effects with machine learning. PLoS One 14, e0208937. 30625159 10.1371/journal.pone.0208937 PMC6326456

[27] Kino S , Hsu YT , Shiba K et al. (2021) A scoping review on the use of machine learning in research on social determinants of health: trends and research prospects. SSM Popul Health 15, 100836. 34169138 10.1016/j.ssmph.2021.100836 PMC8207228

[28] WHO Multicentre Growth Reference Study Group (2006) WHO child growth standards based on length/height, weight, and age. Acta Paediatr Suppl 450, 76–85. 16817681 10.1111/j.1651-2227.2006.tb02378.x

[29] de Onis M , Borghi E , Arimond M et al. (2019) Prevalence thresholds for wasting, overweight and stunting in children under 5 years. Public Health Nutr 22, 175–179. 30296964 10.1017/S1368980018002434 PMC6390397

[30] R Core Team (2006) *R: A Language and Environment for Statistical Computing*. Vienna, Austria: R Foundation for Statistical Computing; available at https://www.r-project.org/ (accessed May 2020).

[31] Kuhn M (2020) Caret: Classification and Regression Training. https://CRAN.R-project.org/package=caret (accessed March 2021).

[32] Mayer ZA & Knowles JE (2015) caretEnsemble: Ensembles of Caret Algorithms. http://CRAN.R-project.org/package=caretEnsemble (accessed March 2021).

[33] Ho TK (1995) Random Decision Forests. Proceedings of 3rd International Conference on Document Analysis and Recognition, Montreal, Quebec, Canada. https://ieeexplore.ieee.org/stamp/stamp.jsp?tp=&arnumber=598994 (accessed March 2021).

[34] Bentéjac C , Csörgő A & Martínez-Muñoz G (2021) A comparative analysis of gradient boosting algorithms. Artif Intell Rev 54, 1937–1967.

[35] Chen T , He T , Benesty M et al. (2018) XGBoost: Extreme Gradient Boosting. R Package Version 0.71.2. https://deepsense.ai/wp-content/uploads/2015/11/xgboost.pdf (accessed March 2021).

[36] Chen T & Guestrin C (2016) XGBoost: A Scalable Tree Boosting System. KDD ’16: Proceedings of the 22nd ACM SIGKDD International Conference on Knowledge Discovery and Data Mining. New York, NY: Association for Computing Machinery. https://dl.cm.org/doi/pdf/10.1145/2939672.2939785 (accessed March 2021).

[37] Clark M (2013) An Introduction to Machine Learning with Applications in R. Notre Dame: Center for Social Research, University of Notre Dame.

[38] Hastie T , Tibshirani R & Friedman J (2009) The Elements of Statistical Learning: Data Mining, Inference, and Prediction, 2nd ed. New York: Springer.

[39] Brownlee J (2016) Machine Learning Mastery with R. Get Started, Build Accurate Models, and Work through Projects Step-by-Step. Melbourne: Machine Learning Mastery Pty.

[40] Landis JR & Koch GG (1977) The measurement of observer agreement for categorical data. Biometrics 33, 159–174. 843571

[41] Motbainor A , Worku A & Kumie A (2015) Stunting is associated with food diversity while wasting with food insecurity among under-five children in east and west Gojjam zones of Amhara region, Ethiopia. PLoS One 10, e0133542. 26285047 10.1371/journal.pone.0133542 PMC4540277

[42] Ethiopian Public Health Institute & ICF (2019) Ethiopia Mini Demographic and Health Survey 2019: Key Indicators. Rockville, MD: EPHI & ICF.

[43] Negash T (2019) Pictures of food insecurity in Amhara region. Res J Soc Sci Manag 9, 39–46.

[44] Khan JR , Chowdhury S , Islam H et al. (2019) Machine learning algorithms to predict the childhood anemia in Bangladesh. J Data Sci 17, 195–218.

[45] Khare S , Kavyashree S , Gupta D et al. (2017) Investigation of nutritional status of children based on machine learning techniques using Indian demographic and health survey data. Procedia Comput Sci 115, 338–349.

[46] Cardoso J , Allwright L & Salvucci V (2016) Characteristics, and Determinants of Child Malnutrition in Mozambique, 2003–11. WIDER Working Paper 2016/147. Helsinki: United Nations University.

[47] Kamiya Y (2011) Socioeconomic determinants of nutritional status of children in Lao PDR: effects of household and community factors. J Health Popul Nutr 29, 339–348. 21957672 10.3329/jhpn.v29i4.8449 PMC3190364

[48] Habyarimana F (2016) Key determinants of malnutrition of children under 5 years of age in Rwanda: simultaneous measurement of three anthropometric indices. Afr Popul Stud 30, 2328–2340.

[49] Poda GG , Chien-Yeh HSU & Chao JC-J (2017) Factors associated with malnutrition among children < 5 years old in Burkina Faso: evidence from the demographic and health surveys IV 2010. Int J Qual Health Care 29, 901–908. 29045661 10.1093/intqhc/mzx129

[50] Akombi BJ , Agho KE , Merom D et al. (2017) Child malnutrition in sub-Saharan Africa: a meta-analysis of demographic and health surveys (2006–2016). PLoS One 12, e0177338. 28494007 10.1371/journal.pone.0177338 PMC5426674

[51] Aheto JMK , Keegan TJ , Taylor BM et al. (2015) Childhood malnutrition and its determinants among under-five children in Ghana. Paediatr Perinat Epidemiol 26, 552–561. 10.1111/ppe.12222 26332093

[52] Masibo PK (2013) Trends and Determinants of Malnutrition among Children Ages 0–59 Months in Kenya (KDHS 1993, 1998, 2003, and 2008–09). Calverton, MD: ICF International.

[53] van den Bold M , Quisumbing AR & Gillespie S (2013) Women’s Empowerment and Nutrition: An Evidence Review. IFPRI Discussion Paper 1294. Washington, DC: International Food Policy Research Institute (IFPRI).

[54] Boah M , Azupogo F , Amporfro DA et al. (2019) The epidemiology of undernutrition and its determinants in children under 5 years in Ghana. PLoS One 14, e0219665. 31365528 10.1371/journal.pone.0219665 PMC6668784

[55] Gurung G (2010) Investing in mother’s education for better maternal and child health outcomes. Rural Rem Health 10, 1352. 20235614

[56] Abubakar A , Uriyo J , Msuya S et al. (2012) Prevalence, and risk factors for poor nutritional status among children in the Kilimanjaro region of Tanzania. Int J Environ Res Public Health 9, 3506–3518. 23202759 10.3390/ijerph9103506 PMC3509468

[57] Sulaiman AA , Bushara SO , Elmadhoun WM et al. (2018) Prevalence and determinants of undernutrition among children under 5-year-old in rural areas: a cross-sectional survey in North Sudan. J Fam Med Prim Care 7, 104. 10.4103/jfmpc.jfmpc_73_17 PMC5958549 29915742

[58] Daoud A , Kim R & Subramanian SV (2019) Predicting women’s height from their socioeconomic status: a machine learning approach. Soc Sci Med 238, 112486. 31470245 10.1016/j.socscimed.2019.112486

[59] Manuel DG , Tuna M , Bennett C et al. (2018) Development and validation of a cardiovascular disease risk-prediction model using population health surveys: the cardiovascular disease population risk tool (CVDPoRT). CMAJ 190, E871–E882. 30037888 10.1503/cmaj.170914 PMC6056289

[60] Allen A , Mataraso S , Siefkas A et al. (2020) A racially unbiased, machine learning approach to prediction of mortality: algorithm development study. JMIR Public Health Surveill 6, e22400. 33090117 10.2196/22400 PMC7644374

[61] Wiemken TL & Kelley RR (2020) Machine learning in epidemiology and health outcomes research. Ann Rev Public Health 41, 21–36. 31577910 10.1146/annurev-publhealth-040119-094437

[62] Morgenstern JD , Buajitti E , O’Neill M et al. (2020) Predicting population health with machine learning: a scoping review. BMJ Open 10, e037860. 10.1136/bmjopen-2020-037860 PMC7592293 33109649

[63] Bagui S , Fang X , Kalaimannan E et al. (2017) Comparison of machine-learning algorithms for classification of VPN network traffic flow using time-related features. J Cyber Secur Technol 1, 108–126.

