# Supplementary material for: Machine learning algorithms for predicting undernutrition among under-five children in Ethiopia
Source: Public Health Nutr. 2021 Oct 8;25(2):269–80. doi: 10.1017/S1368980021004262 (PMC8883776; doi:10.1017/S1368980021004262)
Supplement: Supplementary file 1 [file S1368980021004262sup001.docx]

**Appendix 1** Set of covariates considered as the possible risk factors for childhood undernutrition in Ethiopia.

| -        Child age (< 30 months, 30+ months) |
| --- |
| -        Child size (small, average, large) |
| -        Birth order (1st; 2^nd^; 3rd or later; else=missing") |
| -        Birth interval (Less than 2 years; between 2 and 4 years; greater than 4 years) |
| -        Number of Under-5 children (0-2, 2+) |
| -        Mother’s age (15-19; 20-49) |
| -        Mother’s education (No education, Primary, Secondary/Higher) |
| -        Mother’s marital status (in union, not in union) |
| -        Mother’s nutritional status (BMI) (Underweight, Normal, Overweight) |
| -        Mother’s current work status (working, not working) |
| -        Mother’s contraceptive use and intention (using modern method, using traditional method, non-user – intends to use later, does not intend to use) |
| -        Mother’s occupation (unemployed, Non-manual/professional, agricultural/manual) |
| -        Partner’s education (No education, Primary, Secondary/Higher) |
| -        Partner’s occupation (unemployed, Non-manual/professional, agricultural/manual) |
| -        Family size (Less than 4, between 4 and 8, 8+) |
| -        Total children ever born (0, 1-2, 3-4, 5+) |
| -        Place of residence (rural, urban) |
| -        Wealth index (poor, middle, rich) |
| -        Electricity (present, not present) |
| -        Place delivery (home, health facility, other) |
| -        Region (Tigray, Afar, Amhara, Oromia, Somali, Benshangul, Dire Dawa, Addis Ababa, SNNPR, Gambella, and Harari) |
| -        Toilet facility (improved, not improved) |
| - Cooking fuel (improved, not improved) |
| -        Water source (improved, not improved) |
| -        Religion (Orthodox, Catholic, Protestant, Muslim, Traditional, Other) |
| -        Water source (improved, not improved) |
| -        Has diarrhea (yes, no) |
| -        Postnatal care (yes, no) |
| -        Antenatal care (yes, no) |
| -        Desire for more children (wants within 2 years, wants after 2+ years, wants, unsure timing , undecided, wants no more, sterilized, declared infecund) |
| -        Unmet family planning (yes, no) |
| -        Ever vaccinated (yes, no) |
| -        frequency of reading \|newspaper or magazine (not at all, less than once a week, at least once in a week) |
| -        frequency of listening radio (not at all, less than once a week, at least once in a week) |
| -        Has Tv (yes, no) |
| -        Has radio (yes, no) |
| -        Breastfed in the 1^st^ one hour (Yes, No) |
